# Using Sensors in Organizational Research—Clarifying Rationales and Validation Challenges for Mixed Methods

**DOI:** 10.3389/fpsyg.2019.01188

**Published:** 2019-05-24

**Authors:** Jörg Müller, Sergi Fàbregues, Elisabeth Anna Guenther, María José Romano

**Affiliations:** ^1^IN3 – Universitat Oberta de Catalunya, Castelldefels, Spain; ^2^Universität Klagenfurt, Universitätszentrum für Frauen- und Geschlechterstudien, Klagenfurt, Austria

**Keywords:** mixed methods research, wearable sensors, Bluetooth, indicator, qualitative research, quantitative research, big data

## Abstract

Sensor-based data are becoming increasingly widespread in social, behavioral, and organizational sciences. Far from providing a neutral window on “reality,” sensor-based big-data are highly complex, constructed data sources. Nevertheless, a more systematic approach to the validation of sensors as a method of data collection is lacking, as their use and conceptualization have been spread out across different strands of social-, behavioral-, and computer science literature. Further debunking the myth of raw data, the present article argues that, in order to validate sensor-based data, researchers need to take into account the mutual interdependence between types of sensors available on the market, the conceptual (construct) choices made in the research process, and the contextual cues. Sensor-based data in research are usually combined with additional quantitative and qualitative data sources. However, the incompatibility between the highly granular nature of sensor data and the static, a-temporal character of traditional quantitative and qualitative data has not been sufficiently emphasized as a key limiting factor of sensor-based research. It is likely that the failure to consider the basic quality criteria of social science measurement indicators more explicitly may lead to the production of insignificant results, despite the availability of high volume and high-resolution data. The paper concludes with recommendations for designing and conducting mixed methods studies using sensors.

## Introduction

Sensors are becoming increasingly widespread in social science research. Speculations regarding the potential applications and implications of “big data” are relatively frequent across the entire spectrum of scientific disciplines. However, despite the growing number of debates on the challenges and prospects of big data, and notwithstanding dedicated discussions on “digital methods” (Rogers, 2013) and “digital sociology” (Lupton, 2015; Marres, 2017), more targeted accounts of the methodological implications of using sensors are lacking. This is especially true in the case of mixed methods research (MMR) literature, in which general references to big data do exist (Mertens et al., 2016), but specific engagements from a focused methodological point of view are mostly limited to qualitative contextualization of GPS data (Fielding, 2012; Taylor and Horst, 2013; Remijn et al., 2015).

The fact that MMR debates have failed to systematically address the challenges of the “data deluge” inherent to big data research is unfortunate, since the collection and analysis of sensor data is fundamentally indebted—often without a conscious recognition of such—to key principles and concepts from the MMR field. Drawing upon the MMR literature effectively deepens our understanding of the methodological issues involved in sensor-based research. As a result we contribute to existing knowledge across several disciplines in important ways. First, we alert engineers and computer scientists developing sophisticated models with sensor measures (Fu et al., 2015; Sapru and Bourlard, 2015) to consider the quality of the underlying “raw” data from a social science perspective. Physical sensor measures are too easily equated with social or psychological constructs, thereby underestimating the context-sensitive nature of most social phenomena. Second, we provide organizational researchers concerned with the validation of sensor data (Chaffin et al., 2017; Kayhan et al., 2018) a conceptual framework that situates sensors in relation to established social science research methods. Existing validation efforts combine sensor measures with quantitative as well as qualitative data sources without clearly understanding the inherent limitations of “mixing” methods. MMR debates are here pivotal for adjusting contemporary social science research practice to the new volume and high granularity of sensor-derived data. Third, the present article goes beyond many abstract conceptual reflections regarding “big data” in the social sciences (Tonidandel et al., 2018; Wenzel and Van Quaquebeke, 2018). Our literature review of wearable sensor-based research identifies advantages and disadvantages of different sensor platforms as well as the main social and psychological constructs explored up to date. In combination with our review of the most popular devices this will allow interested scholars to gauge more precisely the relative strengths, weaknesses, and practical implications of each sensor device, for their own research.

In order to advance toward a consistent methodological framework of (wearable) sensors as an instrument for social science research, our argument develops as follows. We suggest first, to clearly separate between two measurement dimensions of sensors, namely the physical data, on the one hand, and the potential social or psychological constructs, on the other hand. On a very basic level, sensors capture changes in the physical environment such as a change of temperature, the relative strength of Bluetooth (BT) signals or the acceleration of objects among others. These physical measures are of little interest to social scientists. Only when (wearable) sensors come to represent social and psychological constructs, do they really constitute an exciting new research instrument. However, as we will argue, the potential fit between the basic physical measures and the higher-level constructs needs to be carefully considered. As our literature review will show, these two levels are often conflated, which has a negative impact on the generation of relevant research insights.

Distinguishing between the physical measurement level and higher-level constructs enables us to focus on the fit between these two levels in a second step. We therefore argue that sensor data, like any other social science indicator, involves three distinct elements: the measurement (or indicator) itself, the unobservable construct to which it refers, and the correspondence between the measurement and the construct (Meyer, 2017). The challenge that needs addressing when using sensors concerns the fact that one and the same physical measurement is expected to represent very different social constructs. Thus, “proximity” between BT enabled devices has been used as an indicator of “friendship,” “advice seeking,” or subjective “well-being” (Sekara and Lehmann, 2014; Matusik et al., 2018; Zhang et al., 2018). It is a key contribution of this paper to show, however, that the quality of fit between the physical measure and the social construct depends to a large degree on how sensor data is combined with additional, complementary data sources. Yet, the crucial methodological question on how to carefully plan for “complementary” data needs at the research design stage with wearable sensors has been largely ignored in the literature.

In a third step we therefore advocate that considering the literature on “mixed methods research” enables a better understanding of the ways in which the quality of fit between the sensor measurement and the targeted social/psychological constructs can be controlled and improved. While the mainstream MMR literature restricts the definition of MMR to the mere combination of qualitative and quantitative data, in this article we use a broader definition of MMR that includes “within-paradigm” combinations. This is, the mixture of several quantitative methods/data as well as the integrating of diverse qualitative methods/data (Morse and Niehaus, 2016; Flick, 2017). At the same time, the MMR literature distinguishes between two broad rationales for “mixing” quantitative and qualitative data, namely “convergent validation” and “complementarity” (Greene et al., 1989; Creswell and Plano Clark, 2007). By reviewing the existing literature on sensor research through the lens of these two MMR rationales, as summarized in Table 1, two important methodological issues start to emerge. First, combining sensor data with other quantitative data sources feeds into “convergent validation” as a result of which the biased nature of sensor measures is revealed. Sensor data in this context is subject to “triangulation” in order to achieve a higher validity of field efforts (Denzin, 1978; Erzberger and Kelle, 2003). Although the potential for validity by convergence has been criticized on various grounds (Fielding and Fielding, 1986; Flick, 2008), and is relatively less frequent in actual research practice (Bryman, 2006), when applied to the context of sensor data, it yields crucial insights into the bias inherent in apparently “raw,” physical sensor measures. Second, important challenges emerge when combining sensor data with qualitative methods, which can follow not only the rationale of “convergent validation” but also “complementarity.” Our analysis of existing approaches of “ethno-mining” (Anderson et al., 2009), “blending” (Bornakke and Due, 2018), or “stitching together” (Blok et al., 2017) sensor data with *qualitative* data shows that the validity of research results is often hampered. This is because the purpose for “mixing” data is left ambiguous, neither being concerned with “convergent validation” nor “complementarity.” By insufficiently conceptualizing the gap between physical measures of behavior and the corresponding layer of social meaning, research efforts fail to deliver significant insights. The negative effects of ignoring the MMR literature for sensor-based research are accentuated by the diverging granularity of the involved data sources. As a further result of our literature review, we argue that the incompatibility between the highly granular nature of sensor data and the static, a-temporal character of traditional quantitative and qualitative data has to date not been sufficiently emphasized as a key limiting factor for sensor-based research.

**Table 1:** Rationales and challenges for integrating qualitative and quantitative data with sensor-based measurements and derived measures.

**Sensor data combined with**	**Rational and purpose of data integration**	**Methods**
+ Quantitative data	Convergent validation of physical sensor measurements	Experimental designs
+ Quantitative data	Convergent validation of higher-level constructs	Tested survey based psychometric scales. Correlation and regression analysis.
+ Qualitative data	Convergent validation of higher-level constructs	Qualitative data to improve the validity of indicators by contextual information
+ Qualitative data	Complementary insights	Ethnographic observations to complement sensor-based findings
+ Qualitative data	Anchoring of data patterns	Ethnographic observations to interpret patterns in sensor data

## Methods

This article grows out of the need to deal with- and reflect upon a concrete research experience with Sociometric badges in R&D teams (Müller, 2018). We agree largely with the growing body of critical literature that fundamental issues regarding construct validity need to be clarified before sensors can fulfill the promise of opening up new and exciting research avenues (Chaffin et al., 2017; Kayhan et al., 2018; Parker et al., 2018). In order to advance toward this goal, we carried out a scoping review of the literature on the use of wearable sensors in organizational research. The methodology for the scoping review followed the steps suggested by Arksey and O'Malley (2005). First, the two research questions guiding the review were identified: What are the strengths and the weaknesses of the different sensor platforms available in the market? What are the main methodological challenges associated with the combination of sensor data with other complementary data sources? Second, the SCOPUS database was searched for relevant publications. The search was performed in the title, abstract and keywords of the publications using terms associated with the concepts of sensor-based research (i.e., wearable sensor^*^, bluetooth, sociomet^*^, sensor-based^*^, wearable comput^*^) and MMR (mixed method^*^). A total of 449 publications were generated from the database search. Third, these publications were screened by title and abstract according to the following three exclusion criteria: (1) health related texts dealing with fitness monitoring, elderly care or injury rehabilitation, (2) purely technical and engineering related articles besides research targeting Human Activity Recognition (HAR) with sensors, and (3) studies related to stationary and centralized sensor systems as deployed in smart-homes. From this screening, 419 references were excluded, reducing the number of references to 30, whose eligibility was assessed by reading the full text of the publications. Using the same exclusion criteria stated above, 7 publications were discarded, leaving 23 references for inclusion in the review. Fourth, data from these references such as the types of sensors used, the challenges addressed by the authors or the validity of the reported measurements, were extracted, charted, and summarized. Additional references were identified by consulting key authors CVs, websites of relevant research groups and the tracking of publications that cite key articles on wearable sensors.

## A Framework for Conceptualizing Sensor Data

The current interest in using sensors for studying social phenomena is embedded in wider debates regarding big data in the social sciences (Savage and Burrows, 2007; Tinati et al., 2014; George et al., 2016; Tonidandel et al., 2018). Sensors constitute one source of big data when tracking, for example, mobility patterns in cities or monitoring health parameters via fitness trackers and smart-phones. Sensors are increasingly pervasive in all aspects of human life. They are widely used in health related applications as already mentioned (rehab, fitness, elderly care, occupational safety) as well as in “smart” cities and homes, tracking of consumer behavior (retail, tourism) or in the social signal processing community (Vinciarelli et al., 2009; Imani et al., 2016; Alcaraz et al., 2017; Goonawardene et al., 2017; Jiang et al., 2017; Oosterlinck et al., 2017). Out of the considerable variety of sensor types and their divergent application fields, the present article concentrates on a relatively well-defined sub-set, namely wearable sensors used in organizational research. Table 2 provides an overview of the most widely used sensor platforms. All these platforms have in common that sensors are usually worn on the body of individual (research) participants as opposed to operating from one (or several) centrally installed, stationary sensor system(s). The platforms differ however in terms of how many individual sensors are integrated into the same system. This has consequences in terms of cost, battery life, size of devices, and data storage. Importantly, the type of sensors or combinations of sensors also conditions which social or psychological phenomena can be measured. Currently, the OpenBeacon badges deployed by the Sociopatterns project (Cattuto et al., 2010) as well as Sociometric badges developed initially by MIT (Olguin et al., 2009; Kim et al., 2012) are the most widely used wearable sensor systems in social science research. Although other technical solutions do exist, their uptake in actual research is rather limited.

**Table 2:** Overview of wearable sensor systems for social science research.

**Sensor platform**	**Sensors**	**Cost per unit**	**Availability**	**Observations**
Sociometric badges	Bluetooth, Infrared, microphone, accelerometer	Approxmately 550 US$	Humanyze, Boston, USA	Proprietary system, including software for data export from badges. High uptake in research community; integrated solution of four sensors; no central hub required. High energy consumption; high cost; data export is “black box”
Sociopatterns/OpenBeacon	RFID	Approxmately 25 US$	Order from openbeacon.org or self-production	Build with open source hardware and software. Low cost, single contact sensor system. Used mostly in healthcare research. Low energy consumption. Central hub for (real-time) location tagging optional. See https://github.com/meriac/openbeacon-ng
Rhythm badges	Bluetooth, microphone	NA	Self-production	Open source hardware and software. This project is currently under active development. So far, no published validation studies are available. See http://www.rhythm.mit.edu/
Smartphone	Bluetooth, microphone, GPS, accelerometer	Cost of modern smartphone	Dependent upon target group	No single solution but constantly changing devices (manufacturer, operating systems) and software apps. Cheap solution due to high smartphone penetration; calibrating/testing physical measures across variety of devices problematic and easily outdated. Location (GPS) and proximity detects simultaneously available. Advantage to combine relatively easily location based measures with call-logs. Custom software install for data collection required
TelosB	RFID	NA	Discontinued	Open source software. System is not available anymore
Hitachi Business Microscope (HBM)	Bluetooth, accelerometer, microphone, infrared, temperature, illumination.	NA	NA	Proprietary system not used outside the Hitachi research. See http://www.hitachi.com/New/cnews/month/2015/02/150209.html
Opo	Ultrasonic wakeup radio	NA	Self-production or order from authors	Open hardware and open source software. Low power and extremely small sensor devices (wearable as adornment). Infrastructure free deployment; relaying of data through smartphone. See https://lab11.eecs.umich.edu/projects/opo/
Multi-sensor board	Microphone, accelerometer, Infrared, visible light, digital compass, temperature, barometric pressure, humidity	NA	Discontinued	Custom build system for research. Listed here as an early system build for social science research goals, but it was not used beyond the initial project described in Wyatt et al. (2008, 2011)

### Measurement Dimensions of Wearable Sensors

In order to provide a solid foundation for assessing the reliability and validity of sensor measures in social- and behavioral research, a distinction between the physical measurement level and the social and psychological constructs needs to be drawn. At the first and fundamental level, sensors measure physical phenomena. This fact, although self-evident, cannot be over-emphasized given the tendency within the literature to conflate the physical sensor measures with social phenomena. BT sensors, for example, produce a quantity called Radio Signal Strength Indicator (RSSI). Values usually range from −40 to −90, where higher numbers indicate a stronger signal which is usually produced by devices being closer together (Liu and Striegel, 2011). Captured with a certain periodicity, RSSI is a “moderate” indicator of varying physical proximity. It is “moderate” because obstacles such as walls can lower the BT signal strength, even though devices are close to each other. Table 3 summarizes the existing literature dedicated to the validation of the physical measurement level of wearable sensors. The more or less stringent fit between the actual measurement and the physical construct it represents is indicated in the “Relational Quality” column in Table 3. As illustrated in the next section, the quality of sensors for certain physical constructs is not as tight as one would expect at this basic level of measurement.

**Table 3:** Overview of sensor types and scientific literature regarding validation of physical constructs.

**Sensor**	**Indicator/measurement**	**Physical construct**	**Relational quality**	**References**	**Sensor platform**
Bluetooth	Radio Signal Strength Indicator (RSSI)	Proximity (and derived network measures)	Moderate	Yu et al., 2016; Boonstra et al., 2017; Chaffin et al., 2017	Sociometric badges
			Moderate	Liu and Striegel, 2011; Boonstra et al., 2017	Smartphone
Infrared	Binary detect	Proximity + orientation of devices (face-to-face; derived network measures)	Moderate	Yu et al., 2016; Chaffin et al., 2017; Müller, 2018	Sociometric badges
RFID	Binary detect	Proximity + obstacles (face-to-face; derived measures)	Moderate	Cattuto et al., 2010; Smieszek et al., 2016; Génois and Barrat, 2018; Elmer et al., 2019	OpenBeacon
Microphone	Volume, frequencies (Hz)	Speech (and derived measures such as speaking duration, turn-taking)	Low	Yu et al., 2016; Chaffin et al., 2017; Chen and Miller, 2017; Kayhan et al., 2018; Müller, 2018	Sociometric badges
Accelerometer	Energy magnitude over three axes	Physical activity	High	Yu et al., 2016; Kayhan et al., 2018	Sociometric badges
Ultrasound	Time-difference of arrival RF / Ultrasound	Proximity + obstacles (and derived network measures)	High	Huang et al., 2014	Opo
GPS	Global coordinates	Physical location	High	<= 0.75 m (95% of the time) U. S. Government GPS.gov	Smartphone & other GPS enabled devices

At a second level, the physical indicator provides the basis for more complex social and psychological constructs. Table 4 lists the higher level concepts that have been addressed so far with wearable sensors. The measurement of such constructs introduces further complexities into the research process, since the quality of research results not only depends upon the quality of the sensor, but also on the degree of consensus around the definition of the social concept under study. How we conceive for example notions of “creativity” or “dominance” on a purely conceptual level is not straightforward but subject to often heated debates within and across the scientific communities (Piffer, 2012). The column “Construct Quality” in Table 4 indicates how disputed the different constructs are. “Stress” for example is relatively well-defined by the level of cortisol in saliva (Taylor et al., 2016). The same holds for “contagion” or “infection,” as defined by the presence or absence of a virus after physical contact. Now the crucial question is to what degree the chosen physical indicators do actually measure these different social concepts. Similar to a medical diagnosis where symptoms are more or less strong indicators of a certain pathology, different sensor-derived measurements are more or less stringent indicators of higher-level constructs. The BT RSSI value is a moderate indicator of physical proximity, which is, in turn, a good indicator of “contagion” but a poor indicator of different types of social relations such as “friendship” or professional “advice.” A microphone, to give another example, provides a numerical record of dominant frequencies (pitch) of the voice which then can be used as an indicator of the persons “sex” (men usually having a lower voice than women) or “stress” (Taylor et al., 2016). Therefore, it is clear that the quality of the overall sensor-based indicator is not just dependent upon the precision of its measurement, but also upon the degree of consensus around the underlying construct and the degree of correspondence between the indicator and the higher-level social- and psychological constructs. Given the variety of constructs for one and the same sensor as described in Table 4, one should expect different levels of validity due to different levels of fit. The fact that we can collect now BT or any other sensor-based data relatively cheaply and with unprecedented detail does not imply that the quality criteria for the measurement properties of these data (i.e., the existence of a solid correspondence between the indicator and the concept it represents) can be ignored.

**Table 4:** Overview of sensor types and scientific literature regarding validation of social and psychological constructs.

**Sensor**	**Indicator/measurement**	**Social and psychological construct**	**Construct quality**	**Relational quality**	**References**	**Sensor platform**
Bluetooth	Radio Signal Strength Indicator (RSSI) and derived (network) measures	Friendship	Moderate	Moderate	Matusik et al., 2018	Sociometric badges
					Eagle et al., 2009; Sekara and Lehmann, 2014	Smartphone
		Advice	Moderate	Moderate	Matusik et al., 2018	Sociometric badges
		Homophily (gender)	High	Moderate	Psylla et al., 2017	Smartphone
		Personality	High	Moderate	Olguin et al., 2009; Lepri et al., 2016	Smartphone
Infrared	Binary detect and derived (network) measures	Personality	High	Moderate	Olguin et al., 2009; Alshamsi et al., 2016	Sociometric badge
		Creativity	High	Moderate	Gloor et al., 2011; Tripathi and Burleson, 2012	Sociometric badge
		Leadership	High	Moderate/Low	Cook et al., 2019	Sociometric badge
		Affect	Moderate	Moderate	Alshamsi et al., 2016; Zhang et al., 2018	Sociometric badge
RFID	Binary detect and derived (network) measures	Contagion (infection, information)	High	High	Vanhems et al., 2013; Barrat et al., 2014	OpenBeacon
					Salathé et al., 2010; Guclu et al., 2016	TelosB
		Homophily (gender)	High	Moderate	Stehlé et al., 2013; Atzmueller et al., 2018	OpenBeacon
Microphone	Volume, frequencies (Hz)	Creativity	High	Moderate	Parker et al., 2018	Sociometric badge
		Stress	High	Low	Martinez Mozos et al., 2016; Taylor et al., 2016	Sociometric badge
		Dominance	High	Moderate/Low	Chen and Miller, 2017; Dietzel et al., 2018	Sociometric badge
		Affect	Moderate	Moderate	Zhang et al., 2018	Sociometric badge
		Talkativeness	Moderate	Moderate	Onnela et al., 2014	Sociometric badge
		Communication	High	Moderate/Low	Holding et al., 2019	Sociometric badge
		Team performance	Moderate/High	Moderate	Wu et al., 2008; Olguin et al., 2009; Dong et al., 2012; Daggett et al., 2017; Endedijk et al., 2018	Sociometric badge
Accelerometer	Energy magnitude over three axes (body activity)	Creativity	High	Moderate/High	Tripathi and Burleson, 2012; Gaggioli et al., 2013; Parker et al., 2018	Sociometric badge
		Dominance	High	Low	Dietzel et al., 2018	Sociometric badge
		Affect / well-being	Moderate	Moderate	Zhang et al., 2018	Sociometric badge
					Yano et al., 2015; Chancellor et al., 2017	HBM
					Doherty et al., 2014	Smartphone
		Social anxiety (mental health)	High	Moderate	Wang et al., 2014; Gong et al., 2019	Smartphone

The following sections will delve into some detail in summarizing the state of the art regarding the validity of sensors on the level of physical measures as well as in relation to higher level constructs. The combination of sensor-based data with qualitative and quantitative research approaches as summarized in Table 1 is key for this next step.

## Combining Sensor Data With Quantitative Methods

Sensor data are often combined with other quantitative data sources and analytic techniques to validate the given metrics. As mentioned, this concerns both the physical measurement level as well as the level of social- and psychological constructs. While the exploration of the former reveals the constructed nature of sensor measures, the latter emphasizes the ambiguities that exist on the level of the social constructs themselves even before sensor devices are deployed for measurement. Work with sensors in the social sciences should not be blinded by a belief in “big data” measurements where the quantity of data is often thought to miraculously compensate for a lack of a well-argued correspondence rule between the social phenomena of interest and the available indicator.

### Scrutinizing “Raw” Sensor Data in the Lab

Much of the excitement around using sensors in the social sciences probably has to do with the promise of providing “objective” measurements of the phenomena under study. Capturing data automatically without direct human intervention addresses the fundamental issue that measurements should not be affected by researchers' bias. As a mechanical form of “observation,” sensors are incorruptible regarding when or what they measure and thus supposedly capable of generating “valid” data that will lead to more solid and far-reaching scientific insights. This promise is even more persuasive within the social sciences where sensors are poised to capture almost imperceptible but elementary behaviors that underlie human interaction. Facilitated by decreasing size and cost, sensors keep track of our behavioral “footprints” on a global scale (Golder and Macy, 2014), underscoring the dominance of numbers as “the modern fact”—simple, unbiased descriptors of phenomena that are only subject to the invariable rules of mathematics (Poovey, 2004, p. xii).

However, although sensors provide a mechanical means of measuring the social, they do not necessarily generate more objective data. Sensors need to be calibrated, have error rates, and are influenced by environmental conditions while data gets sampled, aggregated, and filtered by different algorithms before being exported as “raw” observations for downstream analysis. In fact, as some commentators have remarked, “raw data is an oxymoron” (Gitelman, 2013; Marres, 2017). As shown below, far from being a simple by-product of our activities, sensor-derived data are often an expression of the theories and instruments required for building them in the first place. Therefore, it is increasingly hard to distinguish whether we measure a social phenomenon or the underlying technological devices that mediate it (Marres and Gerlitz, 2015; Marres, 2017). One might ask if we are studying society or technology, RSSI thresholds or knowledge networks, actual friendship ties or algorithmic effects of recommender systems on Facebook? Or, to put it the other way around: to what degree are contemporary social phenomena (such as “friendship”) the effect of technological devices (such as the underlying recommender algorithms in Facebook which suggest new “friends”) rather than genuine social exchange?

The fact that data never “speaks for itself” (Lewis, 2015) has inspired a host of critical studies within behavioral and health sciences, which are summarized in Table 3. These studies are predominantly laboratory experiments where tightly controlled conditions establish the ground-truth on the basis of which sensor-derived measurements are assessed. For instance, Chaffin et al. (2017) strap Sociometric badges onto panels placed into increasing distance to each other in order to see how variability of RSSI metrics correlate with changing physical distance. As the authors report, up to 60% of the variance that BT detects is due to the experimental conditions (physical distance) with 8% of the variance being due to systematic bias of single sensors (Chaffin et al., 2017, p. 9). Next, face-to-face detects are consistently under-reported down to 50% even when placing Sociometric badges in optimal, i.e., manufacturer-specified conditions, for face-to-face detection (Yu et al., 2016; Chaffin et al., 2017). Concerning OpenBeacon sensors, only about half of the actual interactions were recorded by these tested RFID devices (Elmer et al., 2019). Microphones exhibit similar problems: in Yu et al. (2016) sociometers underestimate the duration of speech by 30–40 s while having problems to correctly identify speakers as such (Yu et al., 2016, p. 7). Kayhan et al. (2018) show, through extensive microphone tests, that badges tend to capture changes in volume and frequency accurately, but that differences exist between badges for the same experimental conditions, due to variable sensitivity of each sensor. Building further upon speech detection capacities, turn-taking has a specifically low validity, where sociometers overestimate the ground-truth (actual turns: 6) by a large margin (counted turns: +50) (Chen and Miller, 2017; Kayhan et al., 2018; Müller, 2018). Accelerometer readings provide, on the other hand, more reliable measurements, as reported by both Yu et al. (2016) and Kayhan et al. (2018). On the whole, results are less precise than one would expect at this simple level of physical measurement.

The discrepancy and variability of sensor data with respect to experimental conditions might be further exacerbated by the influence of intermediate processing and data aggregation decisions. Before any data is actually exported for downstream analysis, audio signals are processed by sophisticated algorithms to filter out “noise” from actual “speech” signals—which can substantially alter detected speaking time (Chen and Miller, 2017). Other underlying issues, such as the synchronization of the Sociometric badges internal clock, also play a fundamental role in determining the precision with which badges can measure speaking turns or mirroring activities and, therefore, the extent to which they are able to provide valid measurements. Without a precise synchronization of timestamps between badges, the ability to identify the “same” event across badges is severely impaired (Kayhan et al., 2018).

Thus, although quantitative sensor data enjoy the aura of being “objective” measurements that liberate us from any interpretative effort, upon closer inspection, BT, Infrared, microphone and accelerometer data incorporate a host of technical and measurement biases that need to be taken into account. Instead of providing an exact indicator, it seems more plausible to conceive sensors as probabilistic indicators, even on the level of physical constructs where one intuitively would expect a much more reliable functioning of sensors. This already poses an important limitation to consider before advancing to situations in which sensor data constitute indicators of more complex, social and psychological constructs.

### Convergent Validation I: Social Construct Validity via Quantitative Data

While the basic, critical evaluation of sensor metrics presented during the preceding paragraphs is necessary and certainly has a sobering effect regarding their validity as indicators on a physical level, it only provides a first step in the research process. Combinations with established, quantitative measurement scales also aim to validate sensor-based measurements in relation to higher-level constructs, including “types of social relations” or “creativity,” to name just two (see again Table 4 for further examples). Since validity concerns now go beyond the pure physical construct level, the consensus regarding the underlying (social) construct, as well as the correspondence between the indicator and the construct, do enter into the equation, as the following examples will show.

Given the widespread availability of BT sensors, convergent validation of proximity data is relatively common. Sekara and Lehmann (2014) argue, for example, that by selecting a suitable RSSI threshold (at −80) one can distinguish between strong and weak links that correlate with friendship ties on Facebook (Sekara and Lehmann, 2014). The validation efforts in this case already demonstrate the problematic assumption that social network “friends” are reliable indicators of actual friendship. Matusik et al. (2018), in contrast, use self-reports among leaders in a large-scale research facility to assess the convergent and discriminant validity of BT RSSI thresholds for friendship and advice seeking networks (Matusik et al., 2018). As the authors show, self-reported “friendship” ties correlate to a certain extent with lower RSSI values indicating closer spatial proximity, while advice seeking/receiving ties map best onto more liberal RSSI signals. A further related study concentrates on the correlation between face-to-face detects and derived measures such as the diversity of social communication with positive and negative affect and thus subjective well-being (Alshamsi et al., 2016). Although correlations are found between these two elements, they are quite low (Alshamsi et al., 2016, p. 5). Parker et al., to cite another study, show that the number of speaking segments as measured by Sociometric badges and self-turns in conversations are correlated with higher perceived individual and group creativity as measured by the KEYS survey—“the best-established survey instrument for studying creativity in working environments” (Parker et al., 2018, p. 13).

While these studies provide some evidence regarding the possibilities to map various sensor types of Sociometric badges onto social constructs, they also highlight some critical issues. Sekara and Lehmann rightly contend that “proximity” is a questionable indicator of social relations: “[m]ultiple scenarios exist where people are in close contact but are not friends, one obvious example is queuing” (Sekara and Lehmann, 2014, p. 7). As already discussed, the validity of sensor-derived metrics is not just determined by the measurement precision or the fine-tuning of RSSI thresholds, but it is also framed by the relative strength of the correspondence between the indicator and the chosen construct. Now the crucial point is that in cases where this relationship is rather weak, additional contextual information can improve the quality of the indicator. In this scenario, convergent validation is not only carried out by validating sensor data with other quantitative measurement scales, but also by combining such data with qualitative data sources.

## Combining Sensor Data With Qualitative Methods

Despite the prevalence of the “complementarity” rationale in literature on integration of sensor data with qualitative methods, as Table 1 suggests, other motivations for combining big data with qualitative sources do exist. However, researchers, when reporting their sensor-based findings rarely distinguish between these underlying MMR rationales and consequently underestimate the methodological implications of obtaining well-founded research results. Based upon Table 1, in the following paragraphs, we will describe three clearly distinct rationales for combining qualitative- with sensor-based data. The resulting typology of approaches provides the first in-road to improved research planning using sensors, and improved collection- and interpretation of the obtained data.

### Convergent Validation II: Construct Validity via Qualitative Data

Qualitative data sources can be combined with sensor data for the purpose of validation, i.e., to examine if measurements of the same construct with different instruments converge (or not). A recent exemplary case is available in Parker et al. (2018) where ethnographic observations are used to validate Sociometric speech measurements and body movement in relation to “creativity” in group processes. By closely reading body activity metrics of team members side by side with field notes of the actual working sessions among the environmental scientists, similarities between the two data sources are identified and matched. Thus, specific incidents where the group became “excited and engaged” produce a higher variability in the corresponding body- and speech measurements for the given time slots. Creative moments within the group “are louder on average than any other portion of the group's working day and even louder than their lively lunches and coffee breaks” (Parker et al., 2018, p. 16). Ethnographic observations do not provide complementary insights but rather sufficient contextual detail that make the body- and speech metrics accessible to a specific interpretation—in the current case in terms of a creative flow.

A similar convergent validation logic can be applied to the highly-cited BT studies described in the preceding paragraphs. Eagle et al. (2009) observe, for example, that inferring the friendship network structure through BT signals can be vastly improved when contextual information regarding work and leisure times and places is taken into consideration. Since the ratio of proximity detects outside work (hours) is much higher for friends than for non-friends, “it was possible to predict 96% of symmetric reports of non-friendship and 95% of symmetric friendship” (Eagle et al., 2009, p. 15,275). By observing broad contextual cues of the overall situations under study, qualitative accounts can improve the overall quality of the indicator. Indeed, by providing complementary data on the times and places where “friendship” is more likely to occur, the fit between the indicator and the construct can be improved.

### Qualitative Data for Complementary Insights

Contrary to the “convergent validation” rationale, qualitative data might also be used to complement sensor data, i.e., to examine different facets of the same phenomena by using different (complementary) methods. A recent, exemplary case, is a study published by Bornakke and Due (2018) on the utility of bike signs in the city of Copenhagen. Researchers tracked mobility patterns of cyclists via GPS and combined these with participant observation, direct inquiries and a short questionnaire. The sensor-based information on actual bicycle journeys was thus contextualized with the cyclists' stories explaining their choice of one trajectory or another. The qualitative material explored the “why” question, based upon “what” had happened, i.e., the difference between morning vs. afternoon routes. Or, to put it the other way round, the GPS data “extends the thick observations with knowledge on the generalizability of the behavior of using multiple routes to and from one's home.” (Bornakke and Due, 2018, p. 12).

The way qualitative and sensor data are combined in this example follows a strong “complementarity” rationale where qualitative observations provide a new layer of meaning to the quantitative mobility tracks. This is possible because GPS is a relatively reliable indicator of actual positions in the city; the coordinates are valid as indicators of physical routes and deliver an “autonomous” result to be used for further analysis, questioning and interpretation by researchers. Qualitative data adds here a complementary layer of meaning rather than being preoccupied with untangling the precise social situation represented by the data. Therefore, what this example shows is that only when sensor data has been established as an independent and valid source or measurement, does it make sense to “complement” it with qualitative data. The importance of the dialectic between “complementarity” and “validity” is illustrated more clearly in the example presented in the next section.

### Anchoring Sensor Data via Qualitative Data?

A third approach to combine qualitative data with sensor data does not aim for convergent validation of certain constructs, nor does it provide complementary insights. Rather, it constitutes a more problematic account where qualitative insights aim to make the data interpretable as such. This approach can be illustrated by the following example. In their study at the Danish Technical University, Blok et al. (2017) tracked an entire freshman class of 800 people using BT sensors in smartphones. Ethnographic observations are part of the overall methods, used to contrast the recorded “big” interaction traces with first-person accounts of “thick” descriptions during selected events such as a student party for example. Given that BT is a relatively fuzzy indicator, the analytic work involved probing different aggregation and visualization techniques of the BT signals while constantly verifying the corresponding time slots with the ethnographers' descriptions. Digital data, as the authors explain, “allow for great plasticity” requiring a constant oscillation between extracting “interpretable occasions” from the data and cross-checking with observational accounts (Blok et al., 2017, p. 6). However, what Blok et al. identify as the “great plasticity” of digital data, is nothing but a lack of specificity and hence low quality of proximity-based indicators for social phenomena. In Blok's research, it is the fuzzy nature of proximity data that leads researchers into an endless regress of probing different aggregations levels, filters or visualizations in a hunt for significant patterns of behavior. Exploring the data without any fixed social concept in mind, these patterns mostly fail to emerge because “proximity” in itself is a very ambiguous indicator that can signify anything and nothing. When a distinct proximity/distance pattern finally does emerge and can be correctly labeled with the help of ethnographic field notes—as people leaving the party through a tunnel, at the end of which they “hug goodbye” (Blok et al., 2017, p. 8)—it is not clear that this insight contributes to the wider research question. Although satisfying to “see” this pattern of behavior in the data, it is, somehow, a rather shallow research result because it simply mirrors a broad contextual observation of the event, namely the end of the party. As Doreian and Conti (2012) argue, it is a common mistake of researchers to conflate organizational or spatial context variables with genuine social structures and individual preferences. Without a clear construct that sensor data is supposed to measure, its decontextualized nature leads to interpretations that mirror more the resources and capabilities of the observers than genuine patterns of social behavior.

Therefore, combining qualitative data sources with sensor data is unproductive when the two data sources are simply “stitched” together, without clear theoretical motivation. Although this statement is understandable from an ethnographic and inductively-based approach to data collection, it causes problems when applied to sensor data. And there is a precise reason for that, which will be largely discussed in the next section: inductively exploring sensor data easily falls prey to reproducing broad contextual observations that are first and foremost an expression of the limited observational capacities of the ethnographer rather than an expression of the inherent patterns in high-resolution, time-based sensor data.

## Validation and the Problem of “Matching the Resolution”

The preceding examples of combinations of sensor data with quantitative and qualitative data sources reveal an underlying, but nevertheless important, problem of mismatch between the scale (or resolution) of the sensor data and that of more conventional data collection methods. BT sensors register interaction between persons on a continuous basis. Monitoring a group of 11 people over 5 days can produce up to +100,000 detects. Although data collection methods such as participant observations, interviews, or questionnaires are able to contextualize a number of data points, its focus is, however, limited to events that are more notable and to coarse aggregation levels. Consequently, these methods fail to capture a large part of the continuous temporal information that can be obtained through sensors. Returning to Blok et al. (2017) example, it is clear from this study that the minute details of physical proximity registered by sensors between several hundred participants could not be matched with the observational accounts generated by a well-trained ethnographer. The ethnographic insights in Blok et al. (2017) study (e.g., the “high” and “low” energy of the overall party) remain coarse approximations to the phenomenon under study and, therefore, are ineffective in grasping more subtle events such as, for example, how the overall “energy of the party” emerges out of specific micro-dynamics on the group level. If big data stands for data velocity and volume, qualitative and quantitative accounts have a hard time to keep up the pace. The possibilities of addressing more fine-grained questions are indeed directly dependent on available resources which, most frequently, tend to be limited by “time, research funds, and human coding hours” (Lehmann-Willenbrock et al., 2017, p. 523).

Quantitative, survey-based approaches share a similar fate. The previously cited study by Matusik et al. (2018) ignores the temporal dimension of the proximity data as it correlates *averaged* RSSI values across a 9-day field period with advice- and friendship networks. This high aggregation level could, of course, be broken down into shorter time spans, such as daily- or hourly RSSI mean values. However, the shorter the time-slots over which the sensor data is averaged, the higher the efforts to collect the increasing number of corresponding survey measures. The cited study of Alshamsi et al. (2016) faced similar challenges when participants were asked to respond three times per day to the same questionnaire over a period of 30 days in order to validate their “affect states” with slices of sociometric face-to-face networks. The number of times that these fixed snapshots of affective moods are collected is, to a certain extent, somewhat arbitrary and clearly conditioned by the researchers' ambition to push forward the limits of taxing data collection. What all these validation studies fail to address is the fundamental problem of a mismatch of resolution between sensor-derived data and more traditional, static data types.

As with any measurement instrument, using sensors within the social sciences requires a conscious calibration effort that examines closely how tightly physical- and social constructs are bound to the available sensor metrics. Combining sensor data with other qualitative and quantitative sources confronts the dilemma of either addressing only a fragment of the behavioral measures or having to invest disproportional efforts to collect data with the comparable amount of detail using conventional methods. To say it somewhat figuratively: conventional qualitative and quantitative methods are the bottleneck through which the validation of big data has to pass. This methodological challenge is increasingly being addressed, for example by Luciano et al. (2018) who work toward new approaches to assess “measurement fit” with dynamic, time-based data.

## Concluding Remarks and Recommendations: Toward Mixed Method Research With Sensors

In this article we argue, that in order to reliably use sensor data in organizational research, complementary data needs to be collected. The necessity of integrating sensor data with other, quantitative and qualitative methods sharply contrasts with the predominant tendency in the literature to use (wearable) sensors as a “stand-alone” research instrument (Schmid Mast et al., 2015; George et al., 2016; Tonidandel et al., 2018). The exciting new research questions made possible by the continuous, high resolution monitoring of human behavior remains dependent upon the variable “fit” between physical measurement and the targeted social or psychological constructs. Since the gap between physical sensor data and social construct level is non-negotiable, researchers need to address the validity of their sensor data in the context of their theoretical framework, research questions and by incorporating a mixed methods perspective. As Parker et al. (2018) argue: “From a sociological perspective, this research is intriguing and potentially generative, but its exclusive focus on non-verbal behavior, tendency to use sensors without triangulating methods or confirmatory data, and minimal grounding in sociological theory raise questions about its reliability, validity, and explanatory power.” (p. 9). The following paragraphs provide concrete recommendations for research practice that address the inter-related nature between sensor platform, analytic interest and contextual data needs. We thereby focus on broader methodological considerations that apply to wearable sensors at large; others have provided practical tips when using Sociometric Badges (Chaffin et al., 2017; Kayhan et al., 2018; Parker et al., 2018) or Sociopatterns/OpenBeacons (Elmer et al., 2019) in research. Ethical considerations including privacy issues are discussed in Stopczynski et al. (2014); Metcalf and Crawford (2016).

First, the choice of a sensor platform needs to be adjusted in relation to the research question and a coherent theory that argues for the correspondence between the sensor-based indicator and the relevant constructs. No amount of big data can, by itself, leverage a potential misfit between sensor metrics and higher-level constructs. The overview of Tables 3, 4 provide a first orientation regarding diverse social and psychological concepts explored with different sensor types. “Creativity” for example has been addressed with three types of sensors, namely accelerometers, microphone, and proximity-based sensors (BT or RFID). Researchers interested in exploring “creativity” or other affect-related concepts are well-advised to use an integrated solution such as Sociometric badges or smartphones that can record proximity between participants as well as body activity and speech features. The importance of these data dimensions for monitoring “creative” behavioral markers can justify the relatively higher cost and complex field logistics involved with Sociometric badges (which require daily recharging and data download). On the other hand, researchers interested in monitoring the spread of information or contact and collaboration patterns more generally are well-advised to use simpler systems such as the Sociopatterns/OpenBeacons platform. Although this platform “only” incorporates a proximity sensor, it is the most efficient solution in terms of cost, device size and field logistics for delivering the required “contact” data. Other sensor platforms mentioned in Table 2 cannot be recommended at this point, either because they have been discontinued (TelosB), they are not available for researchers (HBM), or they are still under active development (Rhythm badges).

A second point concerns the recommendations for increasing the validity of sensor measures. As Parker et al. remark, “most current sociometric research conducted by computer scientists and engineers accepts sociometric data at face value” thus risking to create “large datasets and performing sophisticated analysis on data of questionable quality, yielding incomplete or incorrect understandings of small group structure and process” (Parker et al., 2018, p. 25). As the following examples will show, researchers have the choice between different methodological approaches to control the coupling of physical measures, human behavior, and their social interpretation. A tighter coupling of behavior to social meaning is usually dependent upon a strict control of the experimental situation in laboratory settings. More open and complex situations in field research require the recollection of complementary, contextual cues to guarantee the validity of sensor data.

It is important to realize that much of the initial interest surrounding sensors as powerful new research instrument is based upon research carried out in the laboratory. The tight control of the experimental situation eases the interpretative burden of the sociometric data to a considerable degree, fueling hopes for capturing “honest signals” (Pentland, 2008). As sensors can capture a number of elements of body language (i.e., body posture and movement, vocal behavior such as pitch or volume) they provide access to subtle, non-verbal behaviors beyond the literal meaning of words (Hall et al., 2013; Bonaccio et al., 2016). Since these semi-automated behaviors nevertheless steer and structure social interactions on a fundamental level, it becomes conceivable to interpret the physical measurement of behavioral as “honest” signals of social phenomena. Impressive results have been reported by Alex “Sandy” Pentland's group at MIT using Sociometric badges to predict the outcome of speed dating events, elevator pitches, or salary negotiations with surprising precision (see Appendix B of Pentland, 2008, p. 113ff). However, it's worth remembering that the tight coupling of physical sensor measures to social phenomena in these initial studies has been achieved by not only setting up quasi-experimental situations but also by limiting the scope of the dependent variables of interest. By using simple, binary outcome variables (win/lose, trade business card/not, higher salary/lower), relatively high correlations between the sensor measurements and the dependent variables could be achieved. In short, it is the control of “context” that “improves” the validity of the sensor measures as a probabilistic indicator of “honest signals.”

However, as soon as the outcome variables become more complex and the context of social interaction is less defined, theoretical choices regarding the social constructs of interest need to be fined tuned in relation to the type of sensors deployed and their dependency on contextual cues. As soon as sensors are embedded into real-world field settings, the defining feature of “big data”—namely, having a high (temporal) resolution while lacking contextual information (Cai and Zhu, 2015)—comes to the fore. Sensor data is “big” but also “thin” data that needs to be combined with qualitatively grounded “small data” derived from interviews, focus groups or participant observation to unlock their context and hence social meaning (Burrell, 2012; Curran, 2013; Ford, 2014). Eagle et al. (2009) study comes here to mind where the interpretation of physical proximity patterns in terms of “friendship” could be made much more precise by distinguishing between work- or leisure contexts. Complementary observations regarding people's choices when and where to be near each other provides the contextual cues to qualify their physical proximity in terms of specific social relations such as “friendship.” While the observation of context is key for research about “friendship,” complementary observations might be less important when exploring the spread of a contagious disease. In order to study the transmission of a pathogenic germ in a hospital ward, the observation of the physical contact pattern is enough because the validity of the physical proximity measure is a good indicator for contact-related phenomena. Researchers thus have to decide on the relation between the physical measure, the targeted construct and the contextual cues to be observed in order to achieve a better fit and validity of data.

A further example concerns the contextual observations necessary for the study of “creativity.” “Creativity” is usually associated with higher agitation levels in body movement as well as speech (volume) data (Yano et al., 2015; Parker et al., 2018). However, as Parker at al. argue, “having an ethnographer in the room was critical for distinguishing between an exciting scientific episode and a coffee break or photo opportunity” (Parker et al., 2018, p. 25). The observation of the wider context anchors the interpretation of the sensor data in relation to the theoretical interest of the research. The fact that sensor-based methods apparently reduce field efforts in terms of the amount and continuous collection of data needs to be balanced in relation to the additional efforts necessary for contextualizing the collected data. In this regard, under tight research budgets, it is crucial to take into account the resources and skills needed for gathering complementary data by selecting specific events and episodes of observation or for soliciting comments from research participants.

The elaboration of a more precise understanding of observational cues necessary for working with different social and psychological constructs is an important challenge for the future of wearable sensor research. How to best distinguish “knowledge sharing” behavior in proximity data of shared office spaces or small groups for example is such a problem (Génois and Barrat, 2018). From the data it is not directly deducible if the proximity of colleagues is a product of seating order, actual collaboration or even the product of other sources of “bias” such as “people's personality, cultural background, or substance consumption” (Elmer et al., 2019, p. 16). However, addressing validity concerns of sensor data collected in the field is a first, necessary step before considering the true potential of this type of new research instruments, namely to study the temporal dimension of social phenomena (Leenders et al., 2016). Sensors can contribute for example to the study of hitherto marginally explored temporal dimension of “creativity” in organizations by providing a window on the micro-sequencing of events that are responsible of “flow” experiences (Csikszentmihalyi, 1975; Gaggioli et al., 2013). Or, to take the study of leadership emergence as another example: in combination with new analytical Relational Event Models (Butts, 2008), highly granular sensor data enables a much closer look at the “microdynamic relational processes” (Carter et al., 2015) or “micro-origins” (Cook et al., 2019) that govern leadership emergence. Given the predominance of classical, a-temporal accounts of many classical research methods, a vast landscape of sociological and psychological constructs are awaiting to be rethought by organizational researchers in terms of their genuine temporal grounding, including the path dependency between events, their duration, frequencies and cyclicality (Quintane et al., 2013; Ubaldi et al., 2017).

In this article, we have outlined the rather complex processes involved when using wearable sensors in social science research. It counters what is often blind faith in data volume, speed, and variety. As we have argued, the pitfalls of an uncritical acceptance of sensor-based data become apparent by contextualizing the usage of sensor data with MMR rationales as well as basic notions of social science indicators. To the best of our knowledge, the current article is the first to explicitly address issues of wearable sensor research in conjunction with MMR. A careful examination of available sensor types and their corresponding social and psychological constructs as outlined in Tables 3, 4 provides the first steps to a more beneficial deployment of sensors in field research settings. A clear alignment of research question, theoretical constructs and complementary data needs is key for successful wearable sensor research. We hope to have contributed some conceptual clarifications that will provide the framework for a more realistic assessment of these new, existing instruments for social science the potential applications of which are nothing short of exhilarating.

## Author Contributions

JM: conceptual development and first draft of paper. SF: methodological development and major revisions/comments. MR and EG: contribution to conceptual development, empirical fieldwork and analysis of data underlying the paper, and revisions/comments to drafts.

### Conflict of Interest Statement

The authors declare that the research was conducted in the absence of any commercial or financial relationships that could be construed as a potential conflict of interest.
